# Promotion of allergic immune responses by intranasally-administrated nanosilica particles in mice

**DOI:** 10.1186/1556-276X-6-195

**Published:** 2011-03-04

**Authors:** Tokuyuki Yoshida, Yasuo Yoshioka, Maho Fujimura, Kohei Yamashita, Kazuma Higashisaka, Yuki Morishita, Hiroyuki Kayamuro, Hiromi Nabeshi, Kazuya Nagano, Yasuhiro Abe, Haruhiko Kamada, Shin-ichi Tsunoda, Norio Itoh, Tomoaki Yoshikawa, Yasuo Tsutsumi

**Affiliations:** 1 Department of Toxicology and Safety Science, Graduate School of Pharmaceutical Sciences, Osaka University, 1-6, Yamadaoka, Suita, Osaka 565-0871, Japan; 2 Laboratory of Biopharmaceutical Research, National Institute of Biomedical Innovation, 7-6-8 Saito-asagi, Ibaraki, Osaka 567-0085, Japan; 3 The Center for Advanced Medical Engineering and Informatics, Osaka University, 1-6, Yamadaoka, Suita, Osaka 565-0871, Japan; 4 Department of Biomedical Innovation, Graduate school of Pharmaceutical Sciences, Osaka University, 7-6-8 Saito-asagi, Ibaraki, Osaka 567-0085, Japan

## Abstract

With the increase in use of nanomaterials, there is growing concern regarding their potential health risks. However, few studies have assessed the role of the different physical characteristics of nanomaterials in allergic responses. Here, we examined whether intranasally administered silica particles of various sizes have the capacity to promote allergic immune responses in mice. We used nanosilica particles with diameters of 30 or 70 nm (nSP30 or nSP70, respectively), and conventional micro-sized silica particles with diameters of 300 or 1000 nm (nSP300 or mSP1000, respectively). Mice were intranasally exposed to ovalbumin (OVA) plus each silica particle, and the levels of OVA-specific antibodies (Abs) in the plasma were determined. Intranasal exposure to OVA plus smaller nanosilica particles tended to induce a higher level of OVA-specific immunoglobulin (Ig) E, IgG and IgG1 Abs than did exposure to OVA plus larger silica particles. Splenocytes from mice exposed to OVA plus nSP30 secreted higher levels of Th2-type cytokines than mice exposed to OVA alone. Taken together, these results indicate that nanosilica particles can induce allergen-specific Th2-type allergic immune responses in vivo. This study provides the foundations for the establishment of safe and effective forms of nanosilica particles.

## Introduction

With the recent development of nanotechnology, many nanomaterials with innovative functions have been developed. For example, nanoparticles of titanium dioxide and silica have been used in commercial applications related to medicine, cosmetics and food [1]. In particular, amorphous (noncrystalline) nanosilica particles possess extraordinary advantages, including straightforward synthesis, relatively low cost, and easy surface modification [1,2]. Nanosilica particles are increasingly being used for many applications, including cosmetics, food technology, medical diagnosis, cancer therapy, and drug delivery [1-4].

As the use of nanomaterials increases, there is rising concern regarding their potential health risks because there is preliminary evidence that the unique electrical and mechanical properties of nanomaterials is associated with undesirable biological interactions [5,6]. In addition, it has recently become evident that particle characteristics, including particle size and surface properties, are important factors in pathologic alterations and cellular responses [7-10]. For instance, Nishimori et al have previously demonstrated that nanosilica particles with relatively small particle size induce a greater level of toxicity, including liver injury, than do silica particles with larger particle size [11]. To create safe and effective forms of nanomaterials, studies which provide basic information regarding biological responses to nanomaterials are essential.

Numerous studies have shown that several types of nanomaterials increase the incidence of allergic immune diseases [12-14]. Activation of the Th2 response, including production of interleukin (IL)-4, IL-5, and IL-13 from Th2 cells (a subset of CD4^+ ^T cells) and immunoglobulin (Ig) G1 or IgE from B cells, is responsible for many of the pathologic features of allergic immune diseases [15]. Some reports have shown that intranasal or airway exposure to nanomaterials promotes allergic immune responses, indicating the immune-activating potential of nanomaterials [12,13]. However, the role of the different physical characteristics of nanomaterials in the production of allergic responses has not been elucidated.

Here, we examined whether intranasal exposure to nanosilica particles has the capacity to promote allergic immune responses in mice. In addition, we investigated the relationship between the size of silica particles and allergic immune responses.

## Materials and methods

### Silica particles

Amorphous silica particles with a diameter of 30, 70, 300 and 1,000 nm (Micromod Partikeltechnologie, Rostock/Warnemünde, Germany, designated nSP30, nSP70, nSP300 and mSP1000, respectively) were used in this study. The particle numbers of silica particles were 3.5 × 10^13^, 2.8 × 10^12^, 3.5 × 10^10^, or 9.5 × 10^8 ^particles/mg (nSP30, nSP70, nSP300, or mSP1000, respectively). Silica particles were sonicated for 5 min and vortexed for 1 min before use. The size of particles was measured using a Zetasizer Nano-ZS (Malvern Instruments, UK). The mean size and the size distribution of particles were measured by means of dynamic light scattering. We confirmed that the particle size distributions of these silica particles were narrow.

### Mice

Female BALB/c mice were purchased from Nippon SLC (Hamamatsu, Japan) and used at 6 to 8 weeks of age. All of the animal experimental procedures in this study were performed in accordance with the National Institute of Biomedical Innovation Guidelines for the Welfare of Animals.

Exposure protocols and detection of antigen-specific antibody responses by enzyme-linked immunosorbent assay

Female BALB/c mice were intranasally exposed to a 20 μL aliquot (10 μL per nostril) containing 10 μg of ovalbumin (OVA; Sigma Chemical Co, St. Louis, MO, USA) as antigen, plus nSP30, nSP70, nSP300, or mSP1000 at concentrations of 10, 50 or 250 μg/mouse, on days 0, 1, and 2. On day 21, plasma was collected to assess antigen-specific antibody (Ab) responses. Antigen-specific IgG and subclass IgG1 Ab levels were determined by enzyme-linked immunosorbent assay (ELISA). The ELISA plates (Maxisorp, type 96F; Nalge Nunc International, Naperville, IL, USA) were coated with 10 μg/ml OVA and incubated overnight at 4°C. Non-specific Ab binding was minimized by incubating the plates with 4% blocking solution (Block Ace; Dainippon Sumitomo Pharmaceuticals, Osaka, Japan) at 37°C for 2 h. Plasma dilutions were added to the antigen-coated plates and incubated at 37°C for a further 2 h. The coated plates were then washed with PBS containing 0.05% Tween 20 and incubated with a horseradish peroxidase-conjugated goat anti-mouse IgG solution (Southern Biotechnology Associates, Birmingham, AL, USA) at 37°C for 2 h. The color reaction was developed with tetramethylbenzidine (MOSS, Inc., Pasadena, MD, USA), stopped with 2N H_2_SO_4_, and quantitated by measuring OD_450 _minus OD_655 _using a microplate reader. OVA-specific IgE Ab levels in plasma were determined using commercial ELISA kits (Dainippon Sumitomo Pharma, Osaka, Japan).

### Isolation of splenocytes

Spleens were aseptically removed and placed in RPMI 1640 medium (Wako Pure Chemical Industries, Osaka, Japan) supplemented with 10% fetal bovine serum, 50 mM 2-mercaptoethanol and 1% antibiotic cocktail (Nacalai Tesque, Kyoto, Japan). The single-cell suspension of splenocytes was treated with ammonium chloride to lyse the red blood cells, and the splenocytes were washed, counted, and suspended in RPMI medium supplemented with 10% fetal bovine serum, 50 mM 2-mercaptoethanol, 1% antibiotic cocktail, 10 mL/L of 100 × nonessential amino acids solution, 1 mM sodium pyruvate, and 10 mM HEPES to a final concentration of 1 × 10^7 ^cells/mL.

### Antigen-specific cytokine responses

Antigen-specific cytokine responses were evaluated by culturing the splenocytes (5 × 10^6 ^cells/well) in the presence of OVA (1 mg/mL) in vitro. Cells were incubated at 37°C for 72 h. Culture supernatants from in vitro unstimulated and OVA-stimulated cells were analyzed by the Bio-Plex Multiplex Cytokine Assay (Bio-Rad Laboratories, Hercules, CA, USA) according to the manufacturer's instructions. The assay results were read on a Luminex 100 Multiplex Bio-Assay Analyzer (Luminex, Austin, TX, USA). The difference between the mean concentration of cytokines in supernatants from in vitro OVA-stimulated cells and unstimulated cells (background) was then calculated.

### Statistical Analysis

All values are expressed as mean ± SEM. Differences between groups were assessed using analysis of variance followed by Turkey's method.

## Results and discussion

### Antigen-specific IgE Ab responses to silica particles

**Figure 1:**
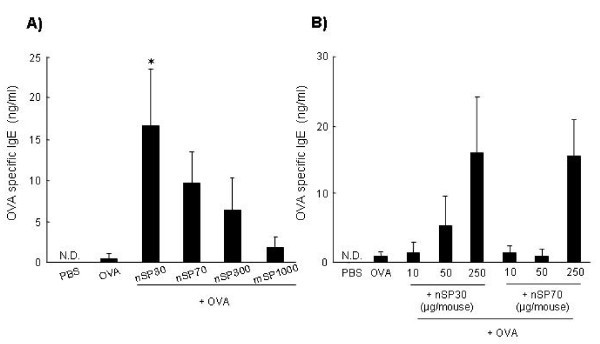
**Plasma OVA-specific IgE Ab responses after intranasal exposure to OVA plus silica particles**. **(a) **BALB/c mice were intranasally exposed to PBS (vehicle control), OVA alone or OVA plus silica particles (250 μg/mouse) on days 0, 1, and 2. **(b) **BALB/c mice were intranasally exposed to PBS (vehicle control), OVA alone or OVA plus the designated dose of nSP30 or nSP70 on days 0, 1, and 2. Plasma was collected on day 21 and analyzed by ELISA to assess (a) the relationship between silica particle size and OVA-specific IgE Ab responses and (b) the dose-response effect of nSP30 and nSP70 on OVA-specific IgE Ab levels. N.D., not detected. Data are presented as mean ± SEM (*n *= 8 to 13; *P < 0.05 *vs *OVA alone).

To assess the relationship between the size of silica particles and allergic immune responses, we used nanosilica particles with diameters of 30 or 70 nm (nSP30 or nSP70, respectively), and conventional micro-sized silica particles with diameters of 300 or 1,000 nm (nSP300 or mSP1000, respectively). The mean secondary particle diameters of the silica particles measured by dynamic laser scatter analysis were 33, 79, 326, and 945 nm, respectively (data not shown). We examined the silica particles by transmission electron microscopy, and confirmed that they were well-dispersed smooth-surfaced spheres (data not shown). To investigate the potential of silica particles to enhance allergic immune responses, we examined their effect on the production of allergen-specific Abs responses in vivo. On days 0, 1, and 2, mice were intranasally exposed to OVA (10 μg/mouse) plus silica particles at concentrations of 10, 50, and 250 μg/mouse. On day 21, we collected plasma from the mice and performed an ELISA to examine anti-OVA IgE Ab responses. The levels of IgE Abs tended to be higher in mice exposed to OVA plus smaller nanosilica particles than in mice exposed to OVA plus larger silica particles (Figure 1a). In particular, the OVA-specific IgE Ab level in OVA plus nSP30-exposed mice was significantly higher than in mice exposed to OVA alone (Figure 1a). We consider that this level of IgE Ab would induce the mast cell degranulation and histamine release, which are major mechanisms underlying anaphylactic reactions in allergic diseases [16]. In addition, the OVA-specific IgE Ab response in mice exposed to OVA plus nSP30 increased in an nSP30-dose-dependent manner (Figure 1b). Taken together, these results suggest that nanosilica particles such as nSP30 are capable of inducing allergic immune responses and have the potential to cause serious allergic symptoms.

### Antigen-specific IgG Abs subclass responses of silica particles

**Figure 2:**
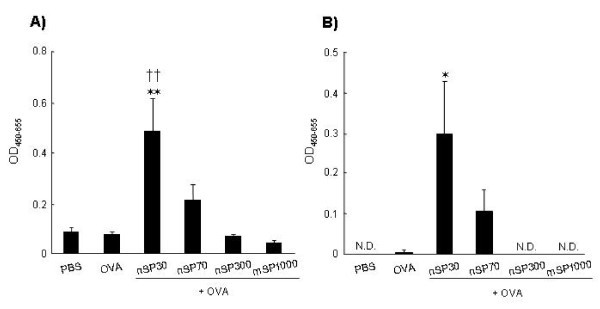
**Plasma OVA-specific IgG and subclass IgG1 Ab response after intranasal exposure to OVA plus silica particles**. BALB/c mice were intranasally exposed to PBS (vehicle control), OVA alone or OVA plus silica particles (250 μg/mouse) on days 0, 1, and 2. Plasma was collected on day 21 and analyzed by ELISA to detect the level of **(a) **OVA-specific IgG and **(b) **OVA-specific IgG1 Ab responses. Data represent mean absorbance at a wavelength of 450 nm (reference wavelength, 655 nm). N.D., not detected. Data are presented as mean ± SEM (*n *= 5 to 8); *P < 0.05, **P < 0.01 vs OVA alone; ^**††**^*P *< 0.01 vs PBS).

Next, to assess the types of immune responses elicited by silica particles, we measured the levels of anti-OVA IgG Ab and anti-OVA IgG1 Ab. IgG1 production is indicative of a Th2-type response. The levels of anti-OVA IgG and anti-OVA IgG1 Abs induced by intranasal-exposure to OVA plus smaller silica particles were higher than those induced by OVA plus larger silica particles (Figure 2); this was similar to the results observed for IgE Ab responses, described above (Figure 1). The levels of OVA-specific IgG Ab and OVA-specific IgG1 Ab in mice exposed to OVA plus nSP30 were significantly higher than in those exposed to OVA alone (Figure 2). These results suggest that nanosilica particles can induce the production of antigen-specific Ab responses including antigen-specific Th2 allergic immune responses.

### Antigen-specific cytokine responses of silica particles

**Figure 3:**
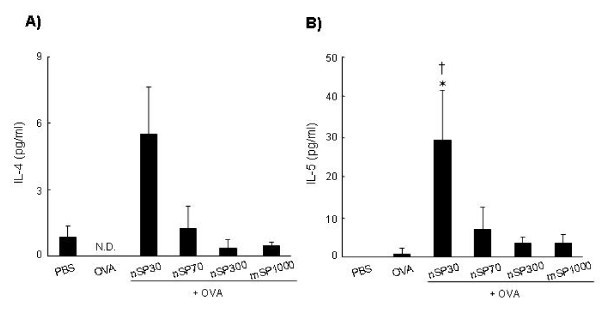
**Cytokine responses induced after intranasal exposure to OVA plus silica particles**. BALB/c mice were intranasally exposed to PBS (vehicle control), OVA alone or OVA plus silica particles (250 μg/mouse) on days 0, 1, and 2. On day 21, splenocytes from each group were prepared and cultured with 1 mg/mL OVA. Culture supernatants were harvested after 3 days of incubation, and the level of OVA-induced IL-4 (A) and IL-5 (B) produced and released into the culture supernatant was analyzed using the Bio-Plex Multiplex Cytokine Assay. Data are presented as mean ± SEM (*n *= 3; *P < 0.05 vs OVA alone; ^**†**^*P *< 0.05 vs PBS).

To clarify the mechanism by which nSP30 elicited an immune response, we analyzed the profiles of cytokines released from splenocytes of OVA-exposed mice. The splenocytes were cultured in the presence of OVA *in vitro*, and the culture supernatants were assessed for Th2-type cytokines by using a multiplexed immunobeads assay. Splenocytes from mice exposed to OVA plus nSP30 exhibited higher levels of Th2-type cytokines (IL-4 and IL-5) than those induced with OVA alone (Figure 3). In contrast, there was hardly any difference in Th1-type cytokine (IFN-γ) production amongst all of the exposed mice (data not shown). In addition, nSP70, nSP300, and mSP1000 did not induce cytokine production (Figure 3). These results suggest that nSP30 nanosilica particle induces a Th2-type immune response in this experiment.

It is not clear why nanosilica particles such as nSP30 would induce Th2-polarized allergic immunity. Our results support previous reports showing that the immune-activating effect of nanomaterials increases with decreasing particle size [12,17]. The mechanisms behind the immune-activating effect of nanomaterials have not been fully elucidated. Nygaard et al [17] showed that the higher specific surface area of nanomaterials as compared to micro-sized particles allows more antigen to be adsorbed per particle. We consider that one possible mechanism by which allergic immune responses induced by nanosilica particles is that many antigen-captured nanomaterials might be taken up by professional antigen presenting cells, such as dendritic cells. Another possible mechanism is that the nanomaterials induce oxidative stress [18,19]. We have observed that nanosilica particles such as nSP30 are stronger inducers of oxidative stress than larger silica particles (unpublished data). Because there is accumulating evidence that oxidative stress plays a role in pro-inflammatory and immune-activating effects [20,21], dendritic cells might be activated more efficiently by nSP30 than by larger silica particles. Furthermore, we also observed that induction of oxidative stress by nanosilica particles is decreased by surface modification of nanosilica particles (unpublished data). Therefore, surface modification might be one approach to decrease allergic immune responses induced by nanosilica particles.

## Conclusion

Here, we show that nanosilica particles have the potential to induce allergic immune responses after intranasal exposure. We consider that further studies of the relationship between the characteristics of nanomaterials and allergic immune responses will facilitate the development of safe and effective nanomaterials.

## Competing interests

The authors declare that they have no competing interests.

## Authors' contributions

TY and YY designed the study; TY, MF, KY, KH, YM and HK performed experiments; TY, YY and MF collected and analysed data; TY and YY wrote the manuscript; HN, KN, YA, HK, ST, NI and TY gave technical support and conceptual advice. YT supervised the all of projects. All authors discussed the results and commented on the manuscript.
